# Additive Re-Manufacturing of Mechanically Recycled End-of-Life Glass Fiber-Reinforced Polymers for Value-Added Circular Design

**DOI:** 10.3390/ma13163545

**Published:** 2020-08-11

**Authors:** Alessia Romani, Andrea Mantelli, Raffaella Suriano, Marinella Levi, Stefano Turri

**Affiliations:** Department of Chemistry, Materials and Chemical Engineering “Giulio Natta”, Politecnico di Milano, Piazza Leonardo da Vinci 32, 20133 Milano, Italy; alessia.romani@polimi.it (A.R.); andrea.mantelli@polimi.it (A.M.); marinella.levi@polimi.it (M.L.); stefano.turri@polimi.it (S.T.)

**Keywords:** additive manufacturing, liquid deposition modeling, composites, glass fibers, end-of-life materials, circular economy, 3D printing

## Abstract

Despite the large use of composites for industrial applications, their end-of-life management is still an open issue for manufacturing, especially in the wind energy sector. Additive manufacturing technology has been emerging as a solution, enhancing circular economy models, and using recycled composites for glass fiber-reinforced polymers is spreading as a new additive manufacturing trend. Nevertheless, their mechanical properties are still not comparable to pristine materials. The purpose of this paper is to examine the additive re-manufacturing of end-of-life glass fiber composites with mechanical performances that are comparable to virgin glass fiber-reinforced materials. Through a systematic characterization of the recyclate, requirements of the filler for the liquid deposition modeling process were identified. Printability and material surface quality of different formulations were analyzed using a low-cost modified 3D printer. Two hypothetical design concepts were also manufactured to validate the field of application. Furthermore, an understanding of the mechanical behavior was accomplished by means of tensile tests, and the results were compared with a benchmark formulation with virgin glass fibers. Mechanically recycled glass fibers show the capability to substitute pristine fillers, unlocking their use for new fields of application.

## 1. Introduction

Nowadays, a great number of end-of-life (EoL) plastic products are continuously being disposed of and less than 30% of this waste is recovered for recycling according to the “European Strategy for Plastics in a Circular Economy” (2018) [1]. However, responsible EoL treatments, which may include reusing, recycling and remanufacturing products can be beneficial from an environmental point of view. Within this framework, the treatment of reinforced polymers represents a key challenge for the European Union, because most composites are currently landfilled, even though this waste management practice is the least preferred by the European Waste Framework Directive [2,3].

In light of the above, the application of the circular economy principles to EoL management of composites is one of the challenges of the modern manufacturing industry [4,5]. The composite market by volume is dominated by glass fiber-reinforced polymers (GFRPs) and one of the manufacturing sectors for GFRPs is the wind energy sector. Wind turbines are characterized by an expected lifetime of approximately 20–25 years. Hence, the amount of wind blade waste across Europe is expected to increase significantly in the coming decades, because of the estimated decommissioning of around 50,000 tons of blades by 2023 [6].

In this perspective, processes such as additive manufacturing are emerging as beneficial for the real development of a circular economy model [7,8]. Additive manufacturing can in fact extend the life of products by fostering customized product part repairing, waste recovering, and recycling [9,10]. Several research studies have been conducted so far for the development of additively manufactured polymer composites [11,12,13]. Very recently, the possibility of reusing mechanically recycled GFRPs in a curable matrix was presented in our previous works [14]. However, the elastic modulus of these 3D printed composites with recycled glass fibers (rGFs) was not comparable to those loaded with virgin glass fibers [15]. Even though the mechanical properties were not high enough because of the low glass fiber content in the recyclates, the potential of 3D printing recycled composites was demonstrated. However, the need to produce low-cost, on-demand, and customized parts and products [16,17], for example in transportation and construction sectors [18,19,20,21], supports the idea of 3D printing of high-performance GFRPs with recycled materials. Therefore, the aim of this work is to show the capability of additive manufacturing to produce GFRPs with good mechanical properties using recycled composites. This was achieved by exploiting a 3D printing process, which enabled the extrusion of a curable resin loaded with mechanical recycled wind blades with a high rGF content.

For the purposes of this paper, the 3D printing technology will be called liquid deposition modeling (LDM) to state that the material in the form of a viscous liquid will be deposited to fabricate a 3D model layer by layer. This technology enables the use of a large material range, which includes ceramics, concrete [22], thermoset polymers, elastomers, and composite materials [11,23,24]. In this work, an LDM technology assisted by UV light (UV-LDM) allows the 3D printing of UV- and thermo-curable resins loaded with mechanically recycled GFRPs obtained by shredding EoL wind blades. 3D printed samples loaded with rGF show good tensile properties, comparable to 3D printed polymers reinforced with virgin fibers. Some design scale models of outdoor furniture were successfully 3D printed by the LDM technology and the materials developed in this study.

## 2. Materials and Methods

### 2.1. Raw Materials

In order to produce the 3D printable ink formulations for a UV-LDM 3D printer, a photo- and thermo-curable acrylic-based resin matrix and virgin or recycled glass fibers were mixed in different percentages. Specifically, the acrylic-based resin, composed of ethoxylate bisphenol A diacrylate resin, was purchased from Arkema, Colombes, France, (local distributor: Came S.r.l., Lainate, Italy), hereinafter named SR349.

A photoinitiator, i.e., ethyl phenyl (2,4,6-trimethyl benzoyl) phosphinate, named TPO-L, provided by Lambson Limited (Wetherby, UK) was also used to trigger the polymerization with the UV light used in the UV-LDM system. Dicumyl peroxide was also added to the mixture to improve the crosslinking degree during the thermal post-curing. In order to compare their behavior, virgin glass fibers or recycled GFRCs were mixed with the resin matrix in different amounts. Milled virgin glass fibers (13 µm nominal diameter and 100 µm nominal length), with the commercial name FIL100, were supplied by Italdry, Briosco, Italy. Recycled GFRCs, hereinafter called GAM Fine recyclate (GAMF), derived from Siemens Gamesa Renewable Energy S.A. EoL wind blades were made of an epoxy resin reinforced by continuous GF. To re-process them with UV-LDM, GFRCs were mechanically shredded to obtain a fine powder by Consiglio Nazionale di Ricerca—Sistemi e Tecnologie Industriali Intelligenti per il Manifatturiero Avanzato (Stiima-CNR), Milano, Italy. All the raw materials were used as received.

### 2.2. Characterization of GAM Fine Recyclate

For a better understanding of the recyclate composition, thermogravimetric analyzes were performed with TA INSTRUMENTS Q500 TGA (TA Instruments, Inc., New Castle, DE, USA). GAM Fine recyclate samples were heated from 25 °C to 800 °C with a 25 °C/min heating rate under an air environment. In this way, the glass content of GAM Fine was evaluated.

Recyclate morphology and fracture surface were evaluated by scanning electron microscopy (SEM). SEM micrographs were obtained using a Cambridge Stereoscan 360 (Cambridge Instrument Company Ltd., Cambridge, UK). Secondary and backscattered electron probes were used, and the sample surfaces were prepared with a physical vapor deposition of gold for 1 min.

SEM micrographs were also analyzed through MATLAB^®^ software (The MathWorks, Inc., Natick, MA, USA). By means of these analyzes, the glass fiber length and diameter of GAMF recyclates were evaluated. The resulting data were then compared with the ones from FIL100 virgin glass fibers datasheet. Accordingly, aspect ratio distributions of the two different fillers were calculated.

### 2.3. 3D Printable Inks Mixing

3D printable ink formulations were optimized for accomplishing printability requirements. The photoinitiator (TPO-L) and the thermal initiator (dicumyl peroxide) with a proportion of, respectively, 3 wt.% and 0.3 wt.% with respect to the total weight of the resin were mixed at room temperature with a magnetic stirrer for 2 h.

FIL100 glass fibers were manually mixed with the resin. GAM fine recyclate was mixed with a double arm kneader mixer (Brabender mixer from C.W. Brabender Instruments, Inc., South Hackensack, NJ, USA) equipped with a rollerblade for 45 min at 40 rpm.

**Figure 1:**
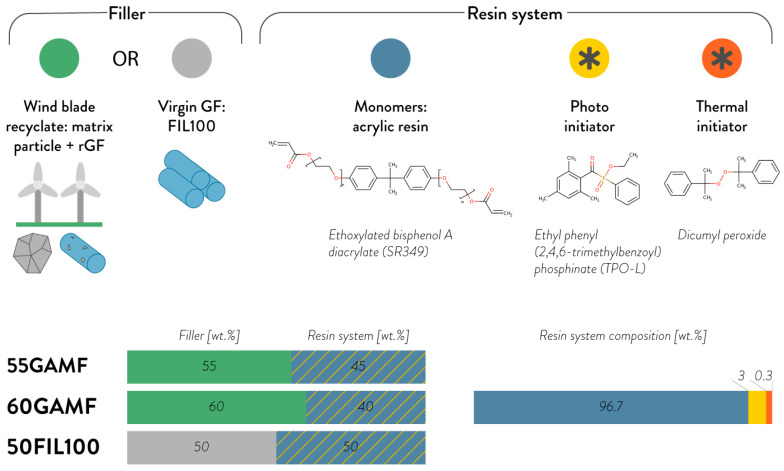
3D Printable ink composition and percentages of the formulations used in this work.

Figure 1 shows the ink composition and the formulations used for this work. From here on, the following nomenclature will be adopted: XZ, where X corresponds to the filler concentration by weight, and Z to the filler short name (FIL100 or GAMF). For instance, 25FILL100 is a formulation with 25 wt.% of FILL100 glass virgin fibers in the ink formulation.

### 2.4. Additive Re-Manufacturing and Post-Curing

The resulting 3D printable inks were then extruded with a modified commercial FDM 3D printer (3Drag) supplied by Futura Group S.r.l., Gallarate, Italy. For obtaining a UV-LDM system, several modifications were made to the 3D printer. Basically, a new extrusion system with an endless screw, a syringe reservoir, and a UV-source were added, as previously described by Romani et al. [14]. In the present work, a 30-mL syringe and a stainless-steel conic UV-shielded nozzle with a diameter of 1.04 mm were equipped to the system for the tests.

Several 3D models were specifically designed for the experimentation by using two different CAD software: Solidworks (Dassault Sistèmes SE, Vélizy-Villacoublay, France) for the tensile specimens and surface finishing samples, and Rhinoceros (Robert McNeel & Associates, Seattle, WA, USA) with “Grasshopper” plugin for the design of scaled 3D models for amusement park environments and outdoor street furniture. For the Gcode creation, Cura open source slicing software (Ultimaker BV, Utrecht, The Netherland) was the most used.

In detail, printings were performed using a speed range between 5 and 10 mm/s and setting a layer height of 0.25 or 0.5 mm. Moreover, the flow percentage varied according to the presence of the filler quantity (from 100% to 105%). According to the specific test, different printing modes were adopted: 100% linear infill for the tensile specimens, or one or two shells with 0% infill for the surface finishing samples and design application scale models, respectively.

As previously mentioned by Mantelli et al. [15], a post-curing cycle is needed to increase the polymerization conversion of the 3D-printed material. In fact, the 3D-printed objects were only partially photo-crosslinked during the 3D printing process. A UV post-curing step was performed with a UV chamber Polymer 500 W (Helios Italquartz S.r.l., Cambiago, Italy) equipped with a UVA emittance mercury vapor lamp type Zs (950 W/m^2^), for 15 min each side. A thermal post-curing step was then carried out in a non-controlled atmosphere oven for 2 h at 140 °C.

### 2.5. Glass Transition Temperature and Gel Content Evaluation

Further analysis was necessary to better understand the degree of cross-linking of the resin matrix. First, the glass transition temperature of the 3D printed materials after the post-curing cycle was evaluated by differential scanning calorimetry (DSC). Tests were performed with a Mettler–Toledo DSC/823e (Mettler Toledo, Columbus, OH, USA) heating ramp from 0 °C to 250 °C with a 20°/min heating rate.

Gel content evaluation was then carried out for determining the total amount of cross-linked material. The obtained material was immersed in acetone with a proportion of 300 mL of solvent for every 2 g of material and was left for 24 h under magnetic stirring. The solution was carefully filtered for collecting residual not solubilized particles in a paper filter (Whatman Filter Papers, Cat No 1001 125), while the solution was poured into the flask. Once the solvent was removed from the filtered solution, the flask and the solid residue inside the paper filter were inserted in a vacuum oven at 50 °C until their weights were constant (approximately after 48 h).

The final weight of the residue inside the flask (solubilized residue) and that inside the paper filter (not solubilized residue) can be easily evaluated knowing the weight of the flask and the paper filter before the process.

Gel percentage (Gel%) was then evaluated with the following equation:

(1) $$\mathrm{Gel}\%=\frac{m_{\mathrm{sample}}-m_{\mathrm{sol}}}{m_{\mathrm{sample}}}\times 100$$

where m**_sample_** is the initial mass of the sample and m**_sol_** is the mass of the solubilized residue.

The mass of the not solubilized residue can be used as a double-check but the recovery of the solid residue can be difficult and part of it can be lost.

### 2.6. Mechanical Characterization

Tensile mechanical properties were evaluated by means of Zwick Roell Z010 (ZwickRoell GmbH & Co. KG, Ulm, Germany) equipped with a 10 kN cell load. ASTM standard test method D3039/D3039M-17 (2017) was followed through the experimentation [25]. 3D printed specimens had a nominal gauge length of 40 mm, a width of 10 mm, an overall length of 100 mm, and a thickness of 2 mm. After the 3D printing, they were manually polished to remove any asperities and to have a constant cross-sectional area along the length of the specimens. The actual dimension was then measured. At least five printed specimens were tested for each formulation at a speed of 1 mm/min.

Halpin-Tsai model for aligned fibers was used to predict the elastic modulus. Glass fibers aspect ratio was employed in the model to obtain an average value from each fiber that was already measured, and it can be seen in the next section.

## 3. Results and Discussion

### 3.1. UV-LDM and Glass Fibers Requirements

In this work, a photo-curable acrylic-based liquid resin filled with two different kinds of GF was considered. By adding a UV source on the extruder head, the resulting 3D printable ink (or material) was able to start its polymerization directly “in situ”, which means immediately after the extrusion, avoiding collapses of the 3D printed structure with UV irradiation (UV-LDM process). Afterwards, the polymerization has to be completed by means of a post-curing treatment developed in the previous work [15].

Mainly, additive manufacturing requirements for UV-LDM may be related to three aspects: the dimension of the filler particles, material rheology, and material reactivity. Since the last two had been already investigated [14], the focus will be on the first requirement.

Limitation in the dimension of the filler particles is related to the extrusion process and the nozzle diameter. Clogging mechanisms of spherical particle-filled liquids have been previously reported from other works [26,27,28]. An equation has been proposed, defining the maximum particle diameter to prevent clogging in FDM systems [29]. Taking into consideration the filler used in this work, the equation developed by Beran et al. [29] could be slightly modified by substituting the particle diameter with the fiber length:

D/l > 6.2,(2)

where D is the nozzle diameter and l the fiber length. As a consequence, to prevent the clogging of the nozzle in this study, the maximum fiber length value is 160 µm with a nozzle diameter of 1 mm.

Many works show evidence of the preferential alignment of the fibers because of shear and extensional flow developing within the nozzle during extrusion [30,31,32]. As a consequence, Equation (2) is quite conservative since it considers that the fibers will pass through the nozzle perpendicular to the flow direction. Nevertheless, since no control on the fiber alignment is present, the value calculated with the modified equation will be considered as a threshold.

**Figure 2:**
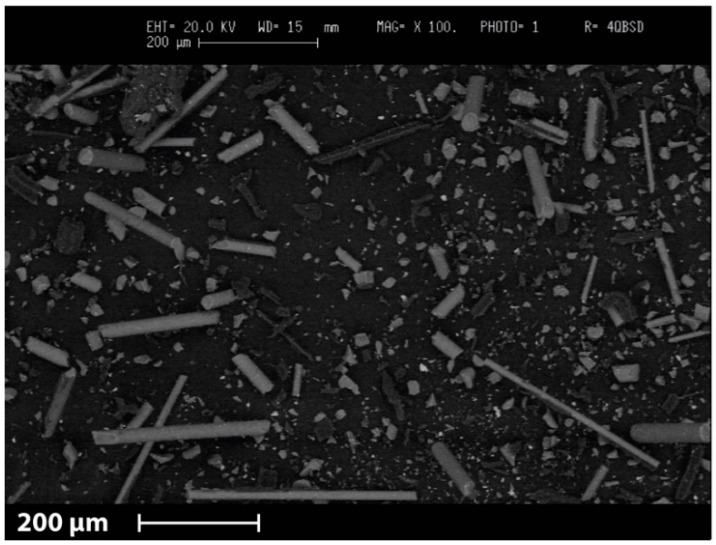
SEM micrograph of GAM fine glass fiber recyclate showing the presence of fibers with different length and/or diameter.

At this purpose, a systematic characterization of the FIL 100 virgin glass fibers and GAM Fine rGFRP was performed. SEM image shows the presence of fibers with different length and/or diameter in GAM Fine recyclate (Figure 2).

**Figure 3:**
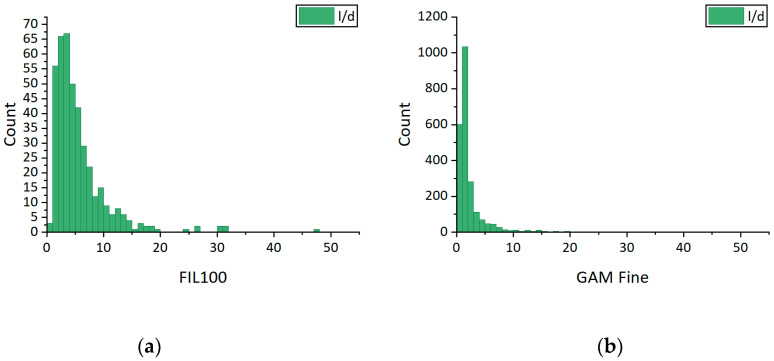
Glass fiber aspect ratio distribution graphs of: (**a**) of FIL 100 virgin glass fibers; (**b**) GAM Fine glass fiber recyclate.

Because of the mechanical recycling, the old matrix is also present in the recycled filler. Consequently, further investigations were performed in order to define the glass content of the recyclate and its fiber length, diameter, and aspect ratio. The numerical values are listed in Table 1, whereas the aspect ratio distribution graphs are visible in Figure 3.

Accordingly, both FIL 100 and GAM Fine recyclate fillers are suitable for the LDM process with a nozzle diameter of 1 mm. Furthermore, GAM Fine recyclate glass content is 70 wt.%, which is a good value for mechanically recycled glass fibers.

### 3.2. Printability and Applications

Very first tests were performed by 3D printing specimens for tensile tests. Specifically, a single formulation of FIL 100 virgin glass fiber (50FIL100) was considered as a benchmark, and two different formulations with GAM Fine recyclate were tested (55GAMF and 60GAMF). Basically, these filler percentages were considered as the best compromise between high filler contents in the inks and their good extrusion during the process.

Further 3D printing trials were then performed. From a printability point of view, the use of the double-arm kneader ensured the homogeneity of the 3D printable ink, decreasing clogging and agglomerates in the formulation. For this reason, the second mixing method was also used in order to create a formulation with a higher recyclate content (60GAMF). A specimen batch was 3D printed with a decreased printing velocity because it was not possible to obtain an extruded filament with a constant diameter with the same printing speed used for the 55GAMF ink. An improved consistency of the extruded filament diameter was obtained also by increasing the layer height (from 0.25 to 0.50 mm). In this way, the process parameters for 60GAMF ink were optimized.

Considering the process parameters, the 55GAMF formulation was the easiest to process. Therefore, other tests were performed to investigate the feasibility and the surface finishing of more complex objects. From literature, material surface finishing investigation is a well-established practice in design fields, relating the physical properties of a material to human perception [33,34,35,36]. Accordingly, material libraries with different material samples properties and finishing have been widely spread, especially for enhancing the development of new applications and materials selection [37]. In this scenario, additive manufacturing methods play a big role in defining surface finishing considering their characteristic layer-by-layer appearance on product surfaces [38].

**Figure 4:**
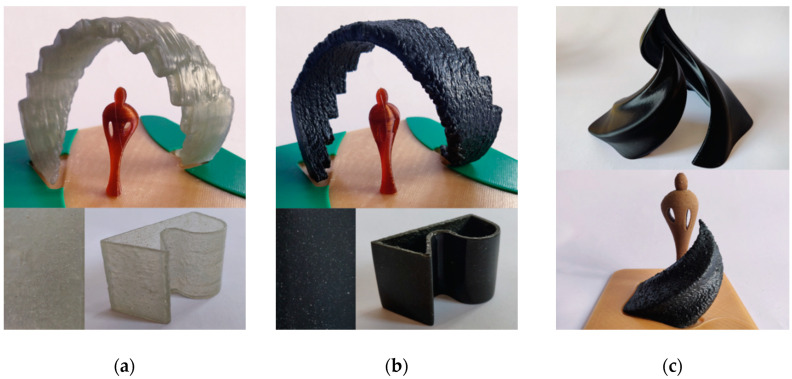
UV-assisted liquid deposition modeling (UV-LDM) additive manufacturing experimentation: (**a**) 1:50 scale model for amusement park application and polished surface finish sample made of 50FIL100; (**b**) 1:50 scale model for amusement park application and polished surface finish sample made of 55GAMF; (**c**) 1:20 scale FDM 3D-printed model of water decoration structures for urban design and amusement applications (top), and 1:20 scale UV-LDM model with 55GAMF (bottom).

For these reasons, a custom 3D model (25 × 50 × 30 mm^3^) was designed for the test. Two different specimens were fabricated with 50FIL100 and 55GAMF inks, respectively. Later, the two samples were manually polished and coated with a transparent gel. Moreover, a printing test with the same formulations was accomplished by using a scale model of a hypothetical building design application in amusement parks. As in the previous work [15], “on-air” 3D printing with a tilt angle of 30° was possible without adding supports. The 3D printed pieces are visible in Figure 4a (50FIL100) and b (55GAMF).

By comparing the 3D printed objects with the two formulations, some considerations on the material aesthetic appearance and surface finishing can be done:

- Virgin glass fiber formulations seem most suitable for color customization by adding pigments directly in the liquid resin system and, consequently, in the 3D printable ink formulation. Furthermore, applications with light and translucency elements can take advantage. On the contrary, air bubbles, voids, and 3D printing defects are more visible compared to rGFRC because of the transparency of the material, and layer-by-layer aspect is clearly noticeable even after the polishing post-processing;
- The rGFRC formulations surface is quite similar to raw stones and granite owing to the dark-grey color and the pattern of the fibers. In addition, a random 3D texture is visible on the surface of the pieces, and the layer-by-layer aspect seems less defined or even eliminated. The opacity and the presence of the texture hide easily voids and defects from view. The polishing post-processing enhances the granite-aspect, eliminating the random texture. As a consequence, these formulations could partially replace outdoor furniture and building applications for free-form or complex shape models.

As a proof-of-concept, a concept of a free-form fountain was designed [14]. A scale model was first 3D printed with a commercial FDM printer. Then, the UV-LDM process with 55GAMF formulation was adopted for the final test, as shown in Figure 4c. Despite further experimentation should be done to develop other 3D printing settings (i.e., retraction), complex high-quality products for real applications could be achieved by scaling up the whole 3D printing system. Moreover, the dark-grey color of the 3D printable ink is not an obstacle to the UV-LDM printing process. This may be related to the scattering effect due to the surface of the fiber particles, that helps to diffuse the UV light. These results demonstrate that rGFRC can be adopted avoiding the virgin GF use.

As mentioned before, these 3D printable materials need 2a UV post-curing and a thermal treatment to increase the degree of crosslinking. To assess the efficiency of crosslinking, the gel content by % gel measurements after post-curing treatments was evaluated for 55GAMF samples.

A % gel value of 99.4% was measured, showing a good degree of cross-linking in 3D-printed objects. Regarding the glass transition temperature, a slight decrease was observed for 55GAMF samples in comparison with the neat resin (from 113.82 °C to 100 °C).

### 3.3. Mechanical Behavior

Mechanical properties play an important role in determining the final application of a specific composite material. This is applicable also for the 3D printable materials presented in this work. Contrarily to virgin fillers, the use of a mechanically recycled material as reinforcement has the intrinsic additional drawback of properties variability due to possible changes from one batch to another. For this purpose, the above-mentioned characterization of GAM Fine (Table 1) was properly made to predict the specific mechanical properties of the developed material.

As previously described, mechanical tests were performed by using a tensile test machine in order to plot the stress versus deformation graph. Accordingly, their experimental elastic moduli, tensile strength, elongation at break and toughness were evaluated.

Several specimen batches were accurately prepared to better understand the effect of process parameters, and material formulation on the mechanical behavior of fillers with a different nature (virgin vs recycled GF). In detail, five different sample batches were considered. The main 3D printing parameters for the different batches are listed in Table 2.

Batches 1 and 2 (Neat Resin and 50FIL100), already presented by Mantelli et al. [15], were used as a benchmark for the mechanical tests. Specifically, specimens of the Batch 1 were produced by casting the neat resin in silicon-based molds, whereas the others were obtained with the UV-LDM process.

Although the higher filler content percentages of the recyclate ink formulations, the virgin GF specimens have a higher value of glass fiber content, according to Table 1 and Table 2. The mechanical behavior comparison is still noteworthy since remarkable results were obtained from the recyclate batches even with lower glass fiber content percentages compared to the virgin GF specimens.

**Figure 5:**
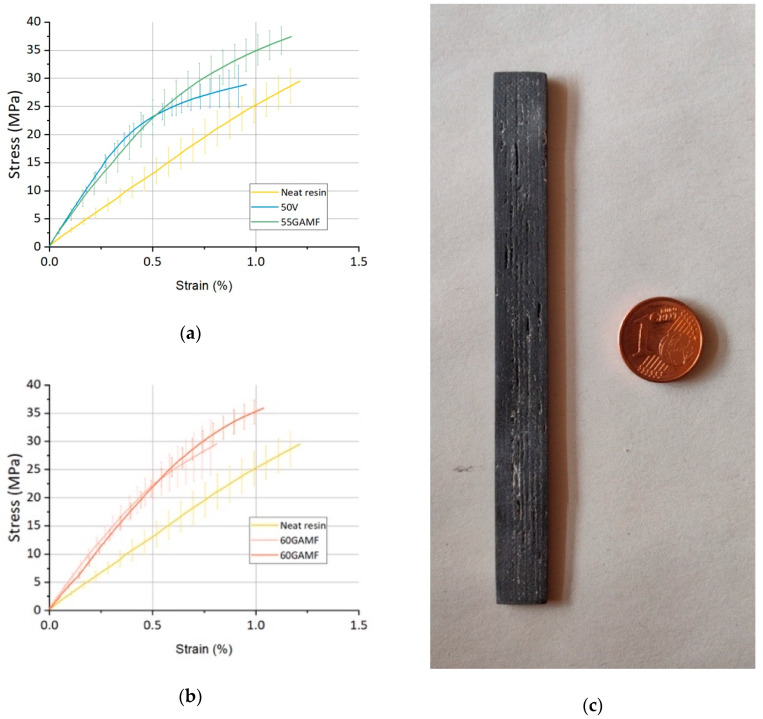
Average stress versus strain graphs with neat resin (Batch 1) as a reference, and specimen image: (**a**) FIL100 (Batch 2) and GAM Fine 3D printable inks (Batch 3), (**b**) GAM Fine 3D Printable inks (Batch 4 and 5), (**c**) GAM Fine 3D printed specimen after manual polishing.

As shown in Figure 5a,b, the overall behavior is similar, and a brittle failure of the samples is observed for each sample batch. The stress versus strain graphs show higher performances for GF reinforced resins. Moreover, bigger areas under their stress-strain curves indicate an enhanced level of toughness due to the GAM Fine recyclate filler presence in the material. A GAM specimen used for the tensile tests is visible in Figure 5c.

The main results from the tensile tests are resumed in Table 3 together with the Halpin-Tsai Model prediction of the elastic moduli, better explained in the next paragraph.

#### 3.3.1. Elastic Properties

Elastic mechanical properties of reinforced composites can be predicted by means of the Halpin-Tsai model [39]. In this way, the reinforcing effect induced by different particle fillers into a homogeneous matrix can be theoretically estimated. The theoretical elastic modulus depends mainly on the geometry of the filler particles, and the stiffness of the matrix and the filler (fibers aspect ratio distribution, fiber elastic modulus, fibers volume fraction, and matrix elastic modulus). Consequently, the above-mentioned characterization of the GF (Table 1) was employed to predict the elastic moduli and the related standard deviation of each specific batch formulation. These data were then compared with the results from the tensile test.

Experimental elastic moduli were evaluated as the line sloped in the linear elastic region for each sample of a specific batch. The mean value was then calculated to obtain a single value and the corresponding standard deviation. The numerical values of the elastic moduli with the standard deviation of the neat resin and the 3D printable materials are shown in Table 3 (Halpin-Tsai Model prediction and the experimental results).

A comparison between the Halpin-Tsai prediction and the experimental results can be done. Considering the experimental results, the Halpin-Tsai Model shows a good prediction of the elastic moduli for the GAM Fine recyclate formulations. On the contrary, this is not true for FIL 100 virgin GF material, which has a significant difference between the prediction and the experimental value. The latter is quite lower than expected. Moreover, its small standard deviation seems to confirm the accuracy of the experimental test and the quality of the 3D printing process.

**Figure 6:**
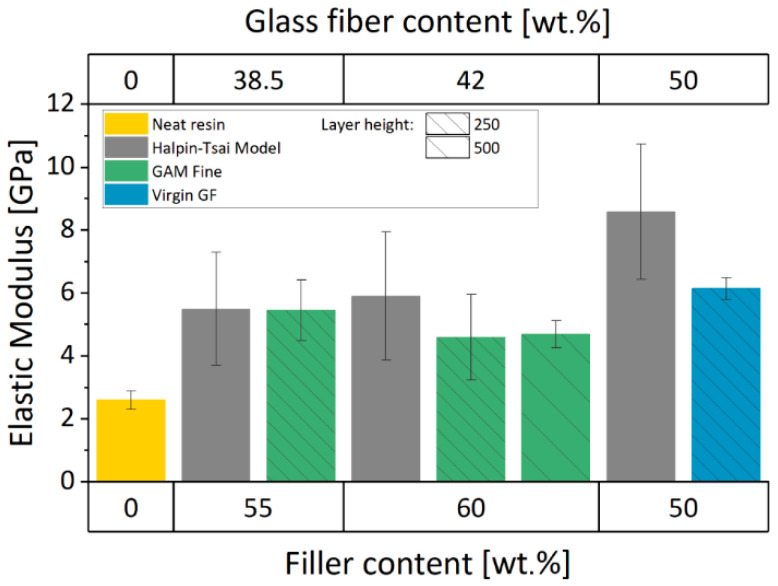
Elastic modulus values for Neat Resin, FIL 100, and GAM Fine-reinforced resins compared with the corresponding Halpin-Tsai Model value.

A two-fold increase in modulus was measured for every 3D printable material formulation when compared to the neat resin. This can be considered a good result, especially for GAM Fine recyclate formulations. As mentioned before, the recyclate filler is composed of shredded glass fibers and the old polymer matrix, which was not previously removed during the mechanical recycling process. Therefore, the GF content in recyclates is lower than in virgin GFs. Even though the amount of GFs in GAM Fine recyclate formulations is lower rather than FIL 100 virgin GF 3D printable inks, they exhibit comparable values of elastic moduli. No significant variations can be observed by increasing the filler percentage content in weight or the layer height used for the printing, instead. The overall comparison is shown in Figure 6.

#### 3.3.2. Failure Properties

From the experimental tensile tests, the main failure properties were also calculated (tensile strength and elongation at break). As previously mentioned, the tested sample batches have not got yield stresses, and their failure mechanism is brittle (Figure 5a,b). Although the noticeable increase of the elastic properties for the virgin and recycled 3D printable materials compared to the neat resin, failure properties show different behavior.

**Figure 7:**
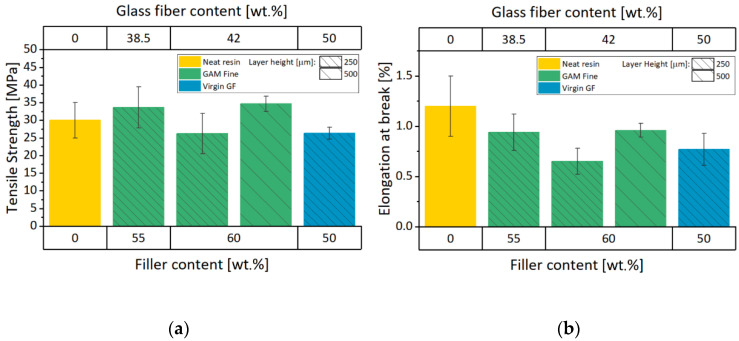
Failure properties of Neat Resin, FIL 100, and GAM Fine-reinforced resins: (**a**) tensile strength graph; (**b**) elongation at break graph.

As highlighted in Figure 7a, the tensile strength of the GAM Fine recyclate formulations is comparable to the neat resin value. This is not true for 60GAMF ink printed at 0.25 mm layer height. In fact, the poorer consistency of the extruded filament diameter led to a decreased strength which may be due to the higher presence of voids. The same trend appears also by considering the elongation at break, confirming the effect of the 3D printing quality on the failure properties.

From Figure 7b, the elongation at break values for all the composite materials are lower than neat resin. As supposed in other works [14,15], this could be attributed to the poor adhesion between the matrix and the filler particles. Moreover, failure properties for recycled GF formulations are better than virgin GF composite except for 60GAMF printed at 0.25 mm layer height. These results could be attributed to a difference in the interfacial properties of the recycled GF when compared to the virgin GF ones as supported by Rahimizadeh et al. [40]. The tensile strength and elongation at break numerical values are resumed in Table 3.

#### 3.3.3. SEM Imaging

After the tensile tests, the fracture surface of the specimens was further analyzed through SEM imaging. Taking into consideration the experimental results, only GAM Fine images are reported for the discussion because of the more performing behavior compared to virgin GF 3D printable material.

**Figure 8:**
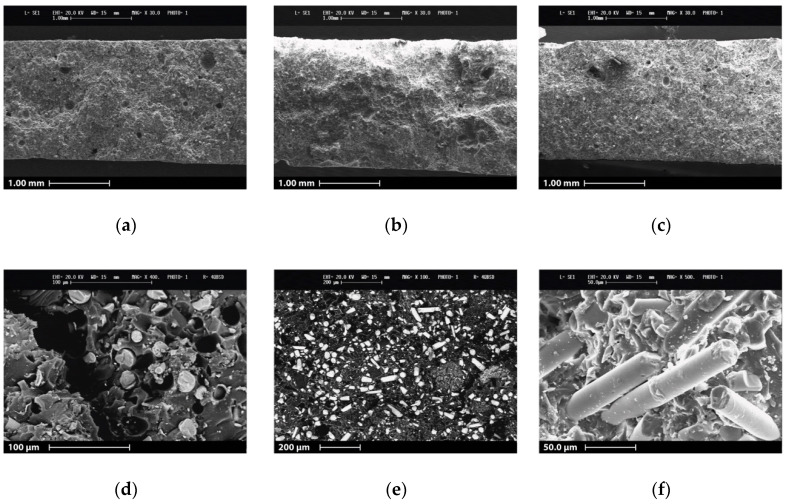
SEM micrographs of GAM fine 3D printed tensile specimen cross-section: **a**) 55GAMF 250 µm layer height; (**b**) 60GAMF 250 µm layer height; (**c**) 60GAMF 500 µm layer height; (**d**) inset image of 55GAMF 250 µm layer height; (**e**) inset image of 60GAMF 250 µm layer height; (**f**) inset image of 60GAMF 500 µm layer height.

Two kinds of images were made for different purposes. Figure 8a–c show the whole fracture surface of specific specimens of 55GAMF (0.25 mm layer height), 60GAMF (0.25 mm layer height), and 60GAMF (0.50 mm layer height), respectively. Their main goal is to evaluate the 3D printing quality of the tested samples. By increasing the magnification, it is possible to check the fiber alignment, their adhesion to the matrix, and specific failure behavior at the fracture surface (Figure 8d–f).

Focusing on the fracture surface SEM images (Figure 8a–c), the microstructure seems quite homogeneous. Furthermore, no visible marks linked to the layer-by-layer 3D printing process are visible, confirming an overall good 3D printing quality of the samples. However, some random voids are clearly visible in the structure. These features are more prevalent in the fracture surface of 60GAMF (0.25 mm layer height, Figure 8b), that shows worse mechanical properties. By changing the layer height from 0.25 mm to 0.50 mm, 60GAMF shows a lower number of voids in Figure 8c. A higher number of voids can be due to increased difficulty with the 3D printing of the formulation with a higher recycled material at equal process parameters. Furthermore, 60GAMF has higher failure properties when it was 3D printed with an increased layer height, and the standard deviation is lower. This demonstrates that a higher 3D printing quality and a more reproducible process can be achieved using optimal 3D printing parameters (0.50 mm of layer height).

In terms of fibers alignment into the matrix, there are no relevant changes for the different GAM Fine filler percentages in the 3D printable ink composition. First, SEM images show a preferential alignment of the fibers (Figure 8e). During 3D printing, the extruded material was deposited parallelly to the tensile test direction, forcing the alignment of the filler particles. As a consequence, fibers are mostly aligned in that direction. This effect has been extensively studied in the literature [31,41], and it could justify the good accordance of the experimental elastic properties to the Halpin-Tsai Model prediction for aligned fibers.

Although the increase of the elastic properties, failure behavior seems not affected by the preferential alignment of the fibers. Figure 8d,f highlight the presence of the fiber pull-out mechanism. The fracture surfaces of the specimens show in fact several detachments of the fibers. Moreover, some fibers are clearly missing (Figure 8d), whereas others are completely undamaged from the tensile tests (Figure 8f). For these reasons, the modest adhesion can be one of the reasons why the neat resin shows higher failure properties than the reinforced 3D printed materials.

#### 3.3.4. Toughness

Further observations can be made considering the areas under the stress–strain curves of the specimen batches. As mentioned before, reinforced GAM Fine recyclate samples present a higher level of toughness compared to the neat resin and the virgin GF 3D printable material (Figure 5a,b). As a consequence, the GAM fine recyclate reinforcement seems to enhance the capability of the 3D printable material to absorb energy and resist before fracture.

**Figure 9:**
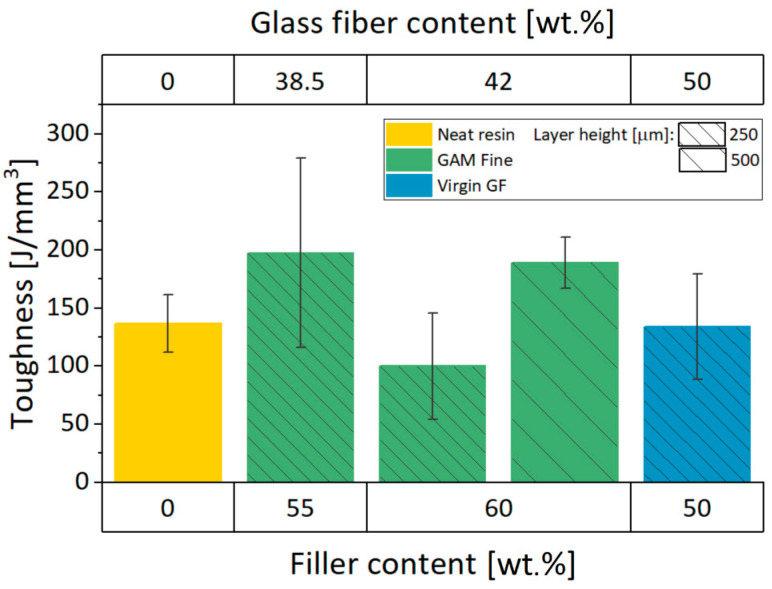
Toughness values for Neat Resin, FIL 100, and GAM Fine-reinforced resins.

In accordance with the elastic and fracture properties previously seen, toughness values show the same trend (Figure 9). Generally speaking, recyclate batches have an increased level of toughness compared to the neat resin, except for 60GAMF (0.25 mm of layer height) batch. 60GAMF (0.50 mm of layer height) has a lower standard deviation also in this case.

Furtherly, there is a remarkable difference between recyclate fillers and virgin glass fibers. 50FIL100 batch toughness is similar to the neat resin value, which means lower toughness than GAM Fine 3D printable materials. For these reasons, toughness may be affected by the 3D printing quality and the filler composition of the 3D printable inks. As a matter of fact, a residual part of the old matrix is still present in GAM Fine recyclate, and it can therefore influence the overall toughness behavior. We can speculate that the presence of the old matrix in the recyclate filler could improve the specific behavior. Consequently, the composite mechanical recycling drawback related to the contamination of the recovered fibers could be overtaken. In fact, it may be considered as an advantage for those applications that need higher toughness values.

## 4. Conclusions

In summary, this work presents the development and investigation of a photo- and thermo- curable 3D printable material for the UV-LDM additive manufacturing process based on recycled GFRC filler. First, a definition of the filler particle requirements for the process was defined. Accordingly, a systematic characterization of the EoL wind blade glass fibers was accomplished.

Formulations containing different filler percentages were 3D printed with different process parameters (layer height and printing speed), and their properties were then compared with a virgin GF 3D printable material taking into consideration printability, material surface aspect, design field of applications, and mechanical behavior. In this way, 3D printable inks with a high amount of rGFRP were processed. As a proof-of-concept, different hypothetical design concept scale models were 3D printed without the use of supports.

Considerable progress has been made about mechanical properties. For the first time in literature, the use of composites with EoL wind blade glass fibers for additive manufacturing applications can be truly considered as a valid alternative to virgin GF fillers. For certain aspects such as tensile strength, their performances could be even better than the pristine GF.

Similarly, new potential design applications for outdoor furniture and building fields were considered owing to the reduced layer-by-layer aspect of the GAM Fine 3D printable material and its raw stone-like and granite visual appearance. Moreover, other fields of application could benefit from the use of the developed materials, such as tooling, automotive, and other BAAM (Big Area Additive Manufacturing) projects, especially in a circular economy perspective.

However, further investigation is needed to overcome the current limitations. A scaling up of the 3D printing layout should be considered for a realistic use of the material in the fields of applications previously mentioned. Furthermore, additional work should be performed to improve the whole UV-LDM process to allow the management of other 3D printing features commonly used during FDM processes (i.e., retraction).

## Figures and Tables

**Table 1:** Glass fiber content (wt.%), mean fiber length (l), mean fiber diameter (d), and aspect ratio (-) of FIL 100 virgin glass fibers and GAM fine glass fiber recyclate (estimated by thermogravimetric analysis).

Filler	Glass Content (wt.%)	Length (µm)	Diameter (µm)	Aspect Ratio (-)
FIL 100	100	73.6 ± 66.5	13	5.7 ± 5.1
GAM Fine	70 ± 1	34.4 ± 47.7	13.5 ± 6.0	2.3 ± 3.2

**Table 2:** 3D Printing parameters for the different tensile specimen batches.

Batch	Filler	Formulation	Filler Content (wt.%)	Glass Fiber Content (wt.%)	Layer Height (mm)	Speed (mm/s)	Raster Angle (°)
1	-	Neat Resin	-	-	-	-	-
2	FIL100	50FIL100	50	50	0.25	20	0
3	GAM Fine	55GAMF	55	38.5	0.25	10	0
4	GAM Fine	60GAMF	60	42	0.25	5	0
5	GAM Fine	60GAMF	60	42	0.50	5	0

**Table 3:** Halpin-Tsai model and experimental elastic modulus, tensile strength, and elongation at break values for the neat resin, FIL 100 and GAM Fine-reinforced resins.

Batch	Filler	Formulation	Layer Height (mm)	Halpin-Tsai Model Elastic Modulus (GPa)	Experimental Elastic Modulus (GPa)	Tensile Strength (MPa)	Elongation at Break (%)
1	-	Neat Resin	-	-	2.6 ± 0.3	30.0 ± 5.0	1.2 ± 0.3
2	FIL100	50FIL100	0.25	8.9 ± 2.2	6.1 ± 0.4	26.3 ± 1.7	0.8 ± 0.2
3	GAM Fine	55GAMF	0.25	5.5 ± 1.8	5.5 ± 1.0	33.6 ± 5.9	0.9 ± 0.2
4	GAM Fine	60GAMF	0.25	5.9 ± 2.0	4.6 ± 1.4	22.8 ± 9.1	0.6 ± 0.1
5	GAM Fine	60GAMF	0.50	5.9 ± 2.0	4.7 ± 0.4	34.6 ± 2.2	1.0 ± 0.1

## References

[1] A European Strategy for Plastics in a Circular Economy. https://ec.europa.eu/environment/circular-economy/pdf/plastics-strategy-brochure.pdf.

[2] Rybicka J., Tiwari A., Leeke G.A. (2016). Technology readiness level assessment of composites recycling technologies. J. Clean. Prod..

[3] Directive 2008/98/EC of the European Parliament and of the Council of 19 November 2008 on Waste and Repealing Certain Directives (Waste Framework Directive). https://eur-lex.europa.eu/legal-content/EN/TXT/PDF/?uri=CELEX:32008L0098&from=EN.

[4] Mativenga P.T., Sultan A.A.M., Agwa-Ejon J., Mbohwa C. (2017). Composites in a Circular Economy: A Study of United Kingdom and South Africa. Procedia CIRP.

[5] Haas W., Krausmann F., Wiedenhofer D., Heinz M. (2015). How Circular is the Global Economy?: An Assessment of Material Flows, Waste Production, and Recycling in the European Union and the World in 2005: How Circular is the Global Economy?. J. Ind. Ecol..

[6] Accelerating Wind Turbine Blade Circularity. https://windeurope.org/wp-content/uploads/files/about-wind/reports/WindEurope-Accelerating-wind-turbine-blade-circularity.pdf.

[7] Despeisse M., Baumers M., Brown P., Charnley F., Ford S.J., Garmulewicz A., Knowles S., Minshall T.H.W., Mortara L., Reed-Tsochas F.P. (2017). Unlocking value for a circular economy through 3D printing: A research agenda. Technol. Forecast. Soc. Change.

[8] Korniejenko K., Łach M., Chou S., Lin W., Mikuła J., Mierzwiński D., Cheng A., Hebda M. (2019). A Comparative Study of Mechanical Properties of Fly Ash-Based Geopolymer Made by Casted and 3D Printing Methods. IOP Conf. Ser. Mater. Sci. Eng..

[9] Ford S., Despeisse M. (2016). Additive manufacturing and sustainability: An exploratory study of the advantages and challenges. J. Clean. Prod..

[10] Rahmizadeh A., Kalman J., Henri R., Fayazbakhsh K., Lessard L. (2019). Recycled Glass Fiber Composites from Wind Turbine Waste for 3D Printing Feedstock: Effects of Fiber Content and Interface on Mechanical Performance. Materials.

[11] Griffini G., Invernizzi M., Levi M., Natale G., Postiglione G., Turri S. (2016). 3D-printable CFR polymer composites with dual-cure sequential IPNs. Polymer.

[12] Mohammadizadeh M., Fidan I., Allen M., Imeri A. (2018). Creep behavior analysis of additively manufactured fiber-reinforced components. Int. J. Adv. Manuf. Technol..

[13] Ferreira I., Vale D., Machado M., Lino J. (2019). Additive manufacturing of polyethylene terephthalate glycol /carbon fiber composites: An experimental study from filament to printed parts. Proc. Inst. Mech. Eng. Part, J. Mater. Des. Appl..

[14] Romani A., Mantelli A., Levi M. Circular Design for Value-added Remanufactured End-of-Life composite material via additive manufacturing technology. Proceedings of the 19th European Roundtable for Sustainable Consumption and Production Circular Europe for Sustainability: Design, Production and Consumption.

[15] Mantelli A., Levi M., Turri S., Suriano R. (2019). Remanufacturing of end-of-life glass-fiber reinforced composites via UV-assisted 3D printing. Rapid Prototyp. J..

[16] Ngo T.D., Kashani A., Imbalzano G., Nguyen K.T.Q., Hui D. (2018). Additive manufacturing (3D printing): A review of materials, methods, applications and challenges. Compos. Part B Eng..

[17] Rayna T., Striukova L. (2016). From rapid prototyping to home fabrication: How 3D printing is changing business model innovation. Technol. Forecast. Soc. Change.

[18] Lim S., Buswell R.A., Le T.T., Austin S.A., Gibb A.G.F., Thorpe T. (2012). Developments in construction-scale additive manufacturing processes. Autom. Constr..

[19] Dixit M.K. (2019). 3-D Printing in Building Construction: A Literature Review of Opportunities and Challenges of Reducing Life Cycle Energy and Carbon of Buildings. IOP Conf. Ser. Earth Environ. Sci..

[20] Moreno Nieto D., Molina S.I. (2019). Large-format fused deposition additive manufacturing: A review. Rapid Prototyp. J..

[21] Post B.K., Chesser P.C., Lind R.F., Roschli A., Love L.J., Gaul K.T., Sallas M., Blue F., Wu S. (2019). Using Big Area Additive Manufacturing to directly manufacture a boat hull mould. Virtual Phys. Prototyp..

[22] Agnoli E., Ciapponi R., Levi M., Turri S. (2019). Additive Manufacturing of Geopolymers Modified with Microalgal Biomass Biofiller from Wastewater Treatment Plants. Materials.

[23] Postiglione G., Natale G., Griffini G., Levi M., Turri S. (2015). Conductive 3D microstructures by direct 3D printing of polymer/carbon nanotube nanocomposites via liquid deposition modeling. Compos. Part Appl. Sci. Manuf..

[24] Postiglione G., Natale G., Griffini G., Levi M., Turri S. (2017). UV-assisted three-dimensional printing of polymer nanocomposites based on inorganic fillers. Polym. Compos..

[25] ASTM D3039/D3039M-17 (2017). Standard Test Method for Tensile Properties of Polymer Matrix Composite Materials.

[26] Shahzad K., D’Avino G., Greco F., Guido S., Maffettone P.L. (2016). Numerical investigation of hard-gel microparticle suspension dynamics in microfluidic channels: Aggregation/fragmentation phenomena, and incipient clogging. Chem. Eng. J..

[27] Agbangla G.C., Climent É., Bacchin P. (2014). Numerical investigation of channel blockage by flowing microparticles. Comput. Fluids.

[28] Sharp K.V., Adrian R.J. (2005). On flow-blocking particle structures in microtubes. Microfluid. Nanofluidics.

[29] Beran T., Mulholland T., Henning F., Rudolph N., Osswald T.A. (2018). Nozzle clogging factors during fused filament fabrication of spherical particle filled polymers. Addit. Manuf..

[30] Bell J.P. (1969). Flow Orientation of Short Fiber Composites. J. Compos. Mater..

[31] Compton B.G., Lewis J.A. (2014). 3D-Printing of Lightweight Cellular Composites. Adv. Mater..

[32] Hmeidat N.S., Pack R.C., Talley S.J., Moore R.B., Compton B.G. (2020). Mechanical anisotropy in polymer composites produced by material extrusion additive manufacturing. Addit. Manuf..

[33] Whitaker T.A., Simões-Franklin C., Newell F.N. (2008). Vision and touch: Independent or integrated systems for the perception of texture?. Brain Res..

[34] Zuo H., Jones M. Exploration into formal aesthetics in design: (material) texture. Proceedings of the 8th International Conference Generative Art.

[35] Miodownik M.A. (2007). Toward designing new sensoaesthetic materials. Pure Appl. Chem..

[36] Karlsson M., Velasco A.V. (2007). Designing for the tactile sense: Investigating the relation between surface properties, perceptions and preferences. CoDesign.

[37] Veelaert L., Ragaert K., Hubo S., Van Kets K., Du Bois E. Bridging design and engineering in terms of materials selection. Proceedings of the Bridging Design and Engineering in Terms of Materials Selection.

[38] Hartcher-O’Brien J., Evers J., Tempelman E. (2019). Surface roughness of 3D printed materials: Comparing physical measurements and human perception. Mater. Today Commun..

[39] Halpin J.C., Kardos J.L. (1976). The Halpin-Tsai equations: A review. Polym. Eng. Sci..

[40] Rahimizadeh A., Tahir M., Fayazbakhsh K., Lessard L. (2020). Tensile properties and interfacial shear strength of recycled fibers from wind turbine waste. Compos. Part Appl. Sci. Manuf..

[41] Shofner M.L., Lozano K., Rodríguez-Macías F.J., Barrera E.V. (2003). Nanofiber-reinforced polymers prepared by fused deposition modeling: Nanofiber-Reinforced Polymers. J. Appl. Polym. Sci..

